# Editorial: The influence of culture and context on managerial leadership

**DOI:** 10.3389/fpsyg.2025.1553980

**Published:** 2025-02-27

**Authors:** André de Waal

**Affiliations:** HPO Center, Hilversum, Netherlands

**Keywords:** culture, managerial leadership, contextual factors, High-Performance Organizations, organizational psychology

In recent years, considerable attention has been devoted to examining the cultural influences on organizational behavior. Within the realm of international business, cross-cultural studies have proliferated, aiming to discern patterns of behavior across diverse organizational settings. However, many of these studies exhibit a tendency to prioritize the universalization of organizational behaviors. This approach often underscores the importance of shared values while offering limited consideration of contextual factors (Hofstede et al., 2010). Moreover, such analyses frequently adopt the managerial perspective as the primary lens, emphasizing the role of leadership in achieving organizational objectives. While insightful, this perspective risks minimizing the distinctiveness of cultural differences by implicitly seeking to homogenize organizational practices (Meyer, 2016).

This Research Topic challenges the prevailing trend of neglecting context in favor of cultural universals. Instead, it advocates for an integrative approach that emphasizes the interplay between culture and context in shaping managerial behavior. Context is not a peripheral concern but a crucial element that, alongside culture, profoundly influences leadership dynamics (Gentry and Sparks, 2012; Nwankwo et al., 2024). To effectively assess managerial performance and evaluate organizational excellence, leaders must consider three critical dimensions: organizational expectations, environmental influences, and their authentic personal perspectives. These dimensions interact in complex ways, creating diverse contexts that demand nuanced managerial approaches. When culture is factored in, the complexity intensifies, as cultural settings vary across multiple axes, including ethnicity, religion, age, and gender (Earley and Peterson, 2014). The interplay of these variables generates a vast array of cultural and contextual combinations, each necessitating tailored leadership strategies.

The concept of High-Performance Organizations (HPOs) is a prime example of the importance of context in managerial leadership. HPO theory identifies universal characteristics necessary for organizational excellence, such as high-quality management, openness, action orientation, continuous improvement, and workforce quality. However, while these characteristics outline what is essential to becoming an HPO, the how of achieving this status varies significantly depending on cultural and contextual factors. High-Performance Managerial Leaders (HPMLs) must adapt these principles to their specific environments, leveraging cultural intelligence and contextual awareness to effectively implement HPO practices (De Waal, 2020). For example, an HPML operating in a collectivist culture may emphasize team cohesion and consensus-building to drive continuous improvement, whereas a leader in an individualist culture might focus on personal accountability and innovation. Similarly, environmental factors such as market dynamics, regulatory frameworks, and societal expectations shape the pathways to becoming an HPO or HPML. Thus, HPO theory highlights that successful adaptation of managerial practices requires not only a deep understanding of these practices but also the ability to tailor these principles to the unique demands of the specific organizational and cultural context.

The four articles in this Research Topic meet those conditions. Han and Han, in their article “*Improving the Service Quality of Cross-Border E-Commerce*”, examine how cultural differences impact consumer perceptions of service quality in cross-border e-commerce. Using Hofstede's cultural dimensions as a framework, the authors found that cultural dimensions significantly impacted consumers' emotional tendencies and service quality perceptions. The study emphasizes the importance of understanding cultural traits to tailor services effectively, highlighting the interplay between context and culture in consumer behavior. This research contributes to the Research Topic by demonstrating how nuanced cultural understanding can enhance organizational strategies in global markets. Dextras-Gauthier et al. explore, in their article “*Organizational Culture and Leadership Behaviors*”, the link between organizational culture, managers' psychological health, and their leadership styles. Their findings reveal that a group-oriented culture positively influences transformational leadership through enhanced managerial wellbeing, while hierarchical cultures are linked to transactional styles. This underscores the critical role of cultural and organizational context in shaping leadership behaviors. This article aligns with the Research Topic by emphasizing how culture-driven psychological resources can facilitate effective leadership. Tanaka et al. validate, in their article “*Motivation to Lead in Japan*” the Motivation to Lead (MTL) scale in a Japanese context, emphasizing the cultural specificity of leadership motivations. Their study reveals how Japanese cultural traits, such as collectivism and high uncertainty avoidance, shape the three MTL dimensions: affective-identity, non-calculative, and social-normative. By highlighting the cultural nuances in leadership development, this research supports the Research Topic's focus on the contextual adaptability of leadership practices. Finally, Nordhall et al., in their article “*Female Managers' Leadership During Telework*”, investigate female managers' experiences during telework, applying the Job Demands-Control-Support model to analyze how demands, control, and support interact in virtual work environments. The findings highlight unique challenges faced by female leaders, such as balancing work-family boundaries and navigating gender biases. Their research contributes to the Research Topic by illustrating how telework context reshapes leadership dynamics and necessitates context-sensitive management strategies.

Recognizing the significance of both culture and context enables a more holistic understanding of organizational psychology. It shifts the focus from a one-size-fits-all paradigm to a more nuanced appreciation of the varied influences that shape managerial behavior (Jackson, 2011). By embracing this perspective, organizations can foster leadership practices that are both culturally sensitive and contextually relevant, ultimately enhancing organizational effectiveness. The editors' hope is that this Research Topic will contribute to this lofty goal.
